# Generative Artificial Intelligence With Youth Codesign to Create Vaping Awareness Advertisements

**DOI:** 10.1001/jamanetworkopen.2025.14040

**Published:** 2025-07-31

**Authors:** Janni Leung, Tianze Sun, Daniel Stjepanović, Giang Vu, Tesfa Yimer, Jason P. Connor, Wayne Hall, Gary C. K. Chan

**Affiliations:** 1 The National Centre for Youth Substance Use Research, School of Psychology, The University of Queensland, St Lucia, Queensland, Australia; 2 Queensland Alliance for Environmental Health Science, The University of Queensland, St Lucia, Queensland, Australia

## Abstract

**Importance:**

Traditional approaches to developing youth vaping awareness campaigns are time-consuming and can create critical delays in public health response. Although generative artificial intelligence (AI) offers promising capabilities for health communication, research has been limited to text-only messages.

**Objective:**

To evaluate (1) the perceived message effectiveness (PME) of AI-generated, youth-codesigned vaping awareness social media advertisements (ads) compared with existing ads from official health agencies and (2) how different source labeling is associated with PME.

**Design, Setting, and Participants:**

This randomized clinical trial used a 2 (ad source) by 4 (source labeling) design and was conducted online from September 2 to September 19, 2024. Participants were individuals aged 16 to 25 years.

**Exposure:**

All participants evaluated 50 ads from 2 sources (within-participants; 25 AI-generated, 25 existing) in random order.

**Main Outcomes and Measures:**

The primary outcome, PME, was measured using the validated PME Scale for Youth, which assessed 2 effects perceptions (vaping perception and behavioral intent) and 3 ad perceptions (attention, information, and convincingness) on 7-point scales, with lower scores indicating better effectiveness for effects perceptions and higher scores indicating better effectiveness for ad perceptions.

**Results:**

Six hundred fourteen individuals (mean [SD] age, 20.5 [2.9] years; 300 female [48.9%]; 300 male [48.9%]; 14 other [2.3%]) provided 30 700 observations. Participants were randomly allocated to 1 of 4 experimentally manipulated labeling conditions (between-participants): (1) no source label (147 participants), (2) made with AI (158 participants), (3) made by the World Health Organization (WHO) (151 participants), or (4) made with AI by the WHO (158 participants). AI-generated ads demonstrated noninferiority to existing ads across all measures. AI-generated ads received better ratings for discouraging vaping (b = 0.09; 95% CI, 0.01 to 0.17), attention-grabbing qualities (b = −0.15; 95% CI, −0.26 to −0.03), and convincingness (b = −0.18; 95% CI, −0.30 to −0.07) (all *P* for noninferiority tests <.001). Source labeling showed no significant association with PME scores (χ^2^ values ranging from 0.10 to 4.19; all *P* > .20).

**Conclusions and Relevance:**

In this randomized clinical trial of vaping awareness social media ads, AI-generated, youth-codesigned ads achieved superior effectiveness ratings compared with existing ads. These findings support the potential for leveraging generative AI technology in public health campaigns, while indicating the need for appropriate governance frameworks as AI-generated health materials become increasingly prevalent.

**Trial Registration:**

ClinicalTrials.gov Identifier: NCT07042789

## Key Points

**Question:**

Can artificial intelligence (AI)–generated vaping awareness advertisements developed through youth codesign achieve effectiveness comparable to that of existing advertisements, and is source labeling associated with their perceived effectiveness?

**Findings:**

In this randomized clinical trial with 614 participants, AI-generated vaping awareness advertisements codesigned with young people achieved perceived effectiveness equal or superior to that of existing advertisements. Source labeling (made with AI vs made by the World Health Organization vs made with AI by the World Health Organization) had no significant association with effectiveness ratings.

**Meaning:**

These findings suggest that codesigned AI-generated health promotion advertisements can outperform traditional campaign materials while maintaining credibility regardless of source attribution, suggesting potential for expediting public health responses to emerging challenges.

## Teaser

This randomized clinical trial evaluates the perceived message effectiveness of artificial intelligence (AI)–generated, youth-codesigned vaping awareness social media advertisements vs existing materials from official health agencies.

## Introduction

Generative artificial intelligence (GenAI) is a new technology with strong potential in health communication.^1,2,3^ Text-based GenAI systems are based on large language models.^4^ On the basis of a user’s prompt, an instruction that steers the GenAI toward the desired outputs, text-based GenAI can produce texts that are indistinguishable from human written content.^5^ Similarly, text-to-image generation systems can generate creative imagery that is on par with those created by human artists, based on users’ prompts that describe the desired scene and style.^6^ GenAI has rapidly gained popularity and been widely adopted across industries, including customer services,^7^ legal practices,^8^ and health care.^1,2,3,5,9,10^

Health communication is a major potential application of GenAI. Mass media campaigns have been the cornerstone in shifting population health behavior.^11,12^ For example, in tobacco control, mass media messaging about the harms of smoking has been central in the World Health Organization’s (WHO) approaches to reducing smoking.^13^ Developing effective campaign messages that resonate with the target audience requires a great deal of time and resources,^1,11^ and there is often a substantial time lag between the emergence of a problem and a response from health agencies. In Australia, research communities and public health advocates have warned about increased youth vaping since early 2018,^14^ but the first mass media campaign warning young people about the harms was only launched in 2021,^15^ missing a key opportunity to educate young people to make informed decisions.

GenAI has the potential to quickly generate individual images and text, which may be useful for developing materials for public health campaigns on social media. For example, when comparing text-only, AI-generated provaccination messages against those from the US Centers for Disease Control and Prevention, the AI-generated messages were perceived to be more effective and evoke more positive attitudes.^5^

Despite the potential of GenAI, 3 key limitations in current research and technology need to be addressed. First, GenAI systems face substantial technical challenges that can impact message quality. These systems are trained on trillions of online texts and text-image pairs. The indiscriminate use of large volumes of data to train these models means that they contain both factual knowledge and misinformation and are then fine-tuned to human preferences.^4^ As a consequence, the messages these systems produce can be seemingly persuasive but factually incorrect, a phenomenon known as *hallucination*.^16^ This limitation is evident in studies examining AI-generated messages, where the outputs have been shown to vary widely in quality, with some being highly effective and others of very poor quality, requiring substantial human editing to correct factual inaccuracies.^5^ In addition, large language models are primarily trained on English text, which means that outputs can be culturally inappropriate and reinforce existing societal biases, ultimately reducing their effectiveness with diverse populations.^17,18,19^ These challenges highlight why a codesign approach with target audiences is essential to ensure that health messages are factually correct, appealing, and engaging to the target audience.

Second, existing studies have focused solely on evaluating text-based messages, yet many contemporary health campaigns used social media advertisements that integrate visuals and text (hereafter referred to as *ads*). It is, therefore, necessary to evaluate more-complex GenAI ads that comprise both text and visual elements.

Third, the majority of studies have shown that messages labeled as AI-generated were rated as less persuasive and trustworthy than those labeled as human-authored.^10,20^ However, despite evidence of this AI discount effect on message persuasiveness, there is limited research on whether this effect can be reduced, for instance, through endorsement by health authorities. This is a critical research question because as GenAI technology matures and becomes mainstream in health communication, it will be essential to understand how to ensure message effectiveness while being transparent about AI authorship.

This study addresses these 3 key limitations through a large-scale evaluation of AI-generated vaping awareness ads that were developed through a 3-phase codesign process with young people. A detailed description of the codesign and development methods is available elsewhere.^21^ In this study, we aimed to test the following hypotheses: First, the perceived message effectiveness (PME) scores for the AI-generated ads codesigned with young people would not be inferior to existing ads created by official health agencies. Second, source labeling would influence PME scores such that ads labeled as AI-generated would receive lower PME ratings compared with those labeled as made by the WHO, but such negative effects of AI labeling would be attenuated when the ads were labeled as AI-generated and endorsed by WHO.

## Methods

The hypotheses and design of this randomized clinical trial were preregistered elsewhere,^22^ and ethics approval was obtained from The University of Queensland. The study was retrospectively registered on ClinicalTrials.gov per the request of the journal editors. The study follows the Consolidated Standards of Reporting Trials (CONSORT) reporting guideline.

### Participants

We recruited participants aged 16 to 25 years old living in Australia through NielsenIQ online panel using a nonprobability sampling approach. Nested quotas were implemented during recruitment to ensure equal representation across age and gender categories. Our sample size has over 95% power for testing the first hypothesis within a 0.5-point inferiority margin and 80% power to detect a moderately small effect size (*F* = 0.13) for the second hypothesis. Data collection took place from September 2 to September 19, 2024. Informed consent was obtained from all participants before the start of the study.

### AI-Generated Ads vs Existing Ads by Official Health Agencies

#### AI-Generated, Codesigned Ads

**Figure 1.**
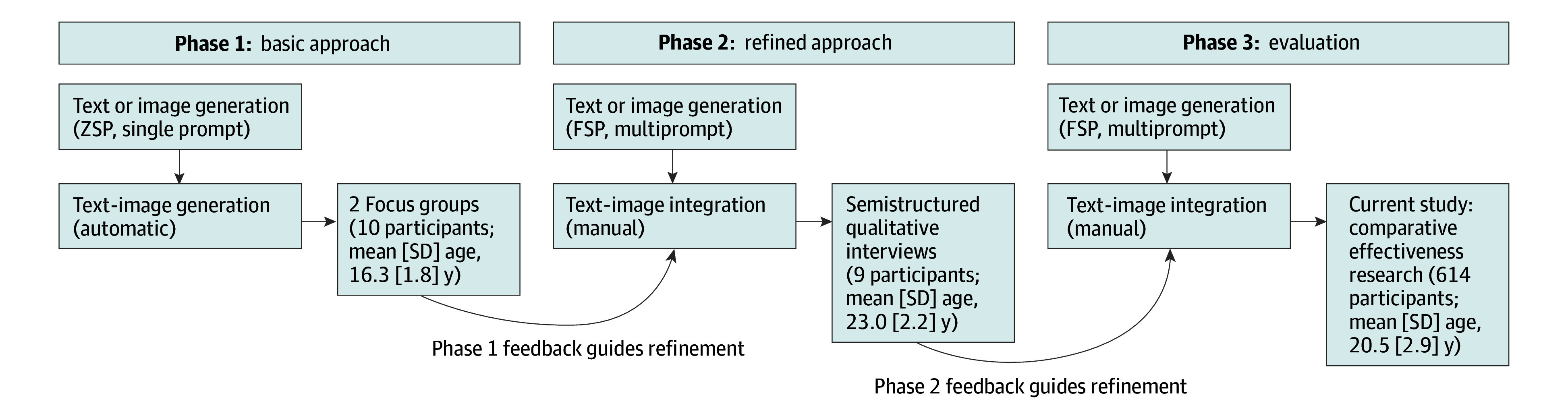
Three-Phase Methods for Developing and Evaluating Artificial Intelligence (AI)–Generated Vaping Awareness Ads Phase 1 used a basic approach: zero-shot prompting (ZSP; model is given a task without any examples) with single prompts (1 prompt is used to interact with the model) and automatic text-image integration. Phase 2 used a refined approach based on phase 1 feedback: few-shot prompting (FSP; model is given a task along with a few examples in the prompt to guide its response) with multiple prompts (multiple sequential prompts are used to refine previous interactions with the model) and manual text-image integration. The current study focuses on phase 3, which evaluated the perceived message effectiveness of AI-generated ads compared with existing ads (N = 614).

We briefly outline the multiphase process for developing the AI-generated, codesigned ads used in this study in Figure 1. The detailed methods of the codesign development process are available elsewhere.^21^ In phase 1, we conducted 2 focus group (10 total participants; mean [SD] age, 16.3 [1.8] years) to evaluate an initial set of 100 AI-generated ads created using a basic approach of zero-shot prompting (the model is given a task without any examples of existing vaping awareness),^23^ single-prompt usage (1 prompt is used to interact with the model), and automatic text-image integration (automatic overlaying of AI-generated text onto corresponding AI-generated images).

On the basis of the feedback from phase 1, we refined our approach for phase 2 to a few-shot prompting approach (the model is given a task along with a few examples in the prompt to guide its response) with multiprompt usage (multiple sequential prompts are used to refine or build upon previous interactions with the model until ads meet quality criteria for accuracy, relevance, and persuasion attempt) and manual text-image integration (text and images) by one of the authors (T.S.) to ensure strong text-image cohesion. Twenty-five AI-generated ads were created using Claude version 3 (Anthropic) for text and Midjourney version 6 (Midjourney, Inc) for images. These refined ads were evaluated through semistructured interviews with 9 participants (mean [SD] age, 23.0 [2.2] years). The feedback from phase 2 interviews was used to further refine the ads in phase 3, resulting in a final set of 25 ads, based on 5 themes: addiction, financial impact, health consequences, industry manipulation, and social norms.

#### Existing Ads From Official Health Agencies

We systematically identified vaping awareness ads from official health agencies using a 2-phase search. First, we searched and identified official health agencies with active antivaping campaigns, including the WHO, US Food and Drug Administration, US Centers for Disease Control and Prevention, and UK National Health Service. Second, we conducted comprehensive searches of their official websites and social media accounts (Facebook, Instagram, and Twitter) using keywords, such as *vaping prevention*, *e-cigarette awareness*, and *youth vaping*.

To ensure consistency and quality, we established strict inclusion criteria for the ads. Ads were included if they (1) were created by an official health agency, (2) were related to 1 of our 5 predetermined themes, (3) were youth-oriented, (4) were in English, and (5) were available as picture files that were in square format or could be cropped to square format.

Twenty-five ads, prioritizing those from WHO and Food and Drug Administration because of their global reach and extensive youth-focused campaigns, were selected. The logos and other descriptors of the health agency that created them were carefully removed. All 50 ads are publicly available elsewhere.^24^

### Procedure

**Figure 2.**
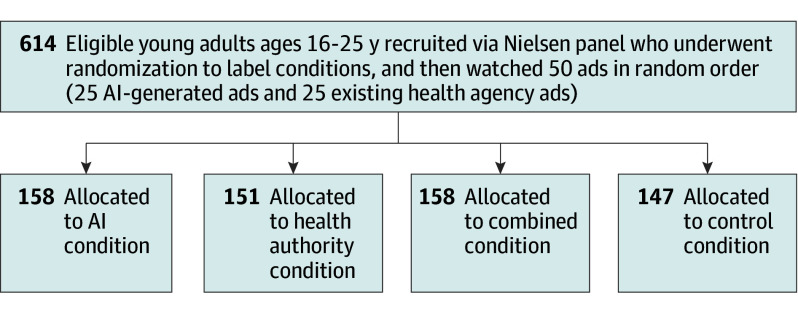
Study Design and Participant Flow Diagram of Experimental Study of Artificial Intelligence (AI)–Generated vs Existing Health Agency Vaping Awareness Ads

This study used a 2 (material source: AI-generated vs existing health agency) by 4 (source labeling conditions: control condition, AI condition, health agency condition, and combined condition) mixed factorial design, with ad source as the within-participants factor and source labeling as the between-participants factor (Figure 2). Participants were randomized into 1 of the 4 source labeling conditions by the experiment platform (Qualtrics) after giving consent and going through screening questions to ensure they were within our target age group. Participants were told the existence of different experimental conditions. Each participant evaluated 50 ads in random order, of which 25 were AI-generated through youth codesign process, and 25 were existing ads from official health agencies.

Participants were randomly assigned to 1 of 4 source labeling conditions: (1) control (ads presented without any source labeling), (2) AI condition (ads labeled with the text “Made with AI”), (3) health agency condition (ads labeled with the text “Made by the World Health Organization (WHO)”), and (4) combined condition (ads labeled with the text “Made with AI, by the World Health Organization (WHO)”). Initially, 911 participants opened the experiment link, 10 participants did not provide consent, 45 were not in our target age group, 32 indicated that they would not commit to providing truthful answers, and 210 did not complete the experiment. Noncompletion was evenly distributed across experimental conditions.

Participants completed the study remotely via an online survey platform, Qualtrics, that was optimized for mobile and desktop viewing. The median (IQR) time to complete the entire experiment was 21 (15-31) minutes.

### Measures

#### Perceived Message Effectiveness

Each ad was rated with 5 items adapted from the validated University of North Carolina Perceived Message Effectiveness (PME) Scale for Youth.^25^ Two items assessed effects perceptions (ie, the ads’ potential to change attitudes and behaviors), and 3 items assessed ad perceptions (ie, judgments about ad characteristics).

The effects perceptions included 2 items: “This ad makes me think vaping is…” (1 = a very bad idea, 4 = neither a good nor a bad idea, 7 = a very good idea) and “This ad…from vaping” (from 1 = strongly discourages me to 7 = strongly encourages me). Lower score indicates stronger effect perceptions. The ad perceptions included 3 items: “This ad grabbed my attention,” “This ad provided useful information,” and “This ad was convincing.” Participants rated these items on a 7-point Likert scale from 1 (strongly disagree) to 7 (strongly agree). Higher scores indicate stronger ad perception.

#### Manipulation Check

At the end of the survey, participants were asked, “Throughout this study, you viewed several health ads about vaping. How were these ads labelled?” Participants selected the response option that best matched what saw: “The ads were labelled as ‘made with AI,’” “The ads were labelled as ‘made with AI, by the World Health Organization (WHO),’” “The ads were labelled as ‘made by the World Health Organization (WHO),’” and “The ads did not have any specific label.” This assessed the effectiveness of our source manipulation.

### Statistical Analysis

We conducted analyses using R statistical software version 4.3.3 (R Project for Statistical Computing) with the lmer^26^ and lmerTest^27^ packages. Linear mixed models were used to test the difference in ratings between AI-generated ads and existing ads. For each PME measure, we fitted 2 models—one with the labeling conditions and one without—and used likelihood ratio tests to assess the overall effect of labeling. Two random intercepts were specified to account for repeated measures from participants and repeated presentation of ads across participants. The noninferiority margins were set at 0.5 points on the 7-point scale because we considered differences smaller than 0.5 points not substantively meaningful. For the second hypothesis regarding labeling effects, we analyzed the full sample and conducted sensitivity analyses with the participants who correctly selected their assigned labeling condition in the manipulation check. Statistical significance was set at 2-sided *P* < .05.

## Results

### Sample Characteristics

Table 1 shows the sample characteristics by labeling condition. Six hundred fourteen individuals (mean [SD] age, 20.5 [2.9] years; 300 female [48.9%]; 300 male [48.9%]; 14 other [2.3%]) provided 30 700 observations. Following random assignment to the 4 labeling conditions (no source label, 147 participants; made with AI, 158 participants; made by the WHO, 151 participants, or made with AI by the WHO, 158 participants), participant characteristics remained well-balanced across demographics, financial status, and tobacco use status.

**Table 1.** Sample Characteristics by Source Labeling Condition

Characteristic	Participants, No. (%)					Effect size^a^
	Total sample (N = 614)	No label (n = 147)	AI label (n = 158)	Health authority label (n = 151)	Combined label (n = 158)	
Gender						
Female	300 (48.9)	77 (52.4)	76 (48.1)	66 (43.7)	81 (51.3)	0.06
Male	300 (48.9)	68 (46.3)	78 (49.4)	82 (54.3)	72 (45.6)	
Other	14 (2.3)	2 (1.4)	4 (2.5)	3 (2.0)	5 (3.2)	
Subjective financial situation^b^						
Do not meet basic expenses	58 (9.4)	13 (8.8)	9 (5.7)	14 (9.3)	22 (13.9)	0.08
Just meet basic expenses	237 (38.6)	58 (39.5)	63 (40.1)	55 (36.4)	61 (38.6)	
Meet needs with a little left	185 (30.1)	46 (31.3)	49 (31.2)	41 (27.2)	49 (31.0)	
Live comfortably	133 (21.7)	30 (20.4)	36 (22.9)	41 (27.2)	26 (16.5)	
Smoking status^c^						
Never used	304 (49.5)	73 (49.7)	76 (48.4)	69 (45.7)	86 (54.5)	0.06
Ever used	309 (50.3)	74 (50.3)	81 (52.6)	82 (54.3)	72 (45.6)	
Vaping status^c^						
Never used	334 (54.4)	81 (55.1)	78 (49.4)	84 (55.6)	91 (57.6)	0.06
Ever used	280 (45.6)	66 (44.9)	80 (50.6)	67 (44.4)	67 (42.4)	
Age, mean (SD), y	20.5 (2.9)	20.8 (2.9)	20.1 (3)	20.6 (2.8)	20.5 (2.8)	0.01

Abbreviation: AI, artificial intelligence.

^a^ Cramer *V* was the effect size measure for the categorical variables; *R*^2^ was the effect size measure for age.

^b^ Participants rated their overall subjective financial situation on a 4-point scale.

^c^ Vaping status was assessed with the question, “How often, if at all, do you currently use vapes/e-cigarettes?” Responses were categorized into never used (participants who selected never used) and ever used (combining all other options, including, “I used to use them, but no longer use,” “I only tried once or twice,” “At least monthly [but not weekly],” “At least weekly [but not daily],” and “Daily”). Smoking status assessed with the question, “How often, if at all, do you currently smoke cigarettes?” with the same categorization as vaping status (never used and ever used).

### PME Ratings

#### Overall Impact of Ads

Table 2 presents the descriptive statistics for the 5 PME measures across labeling conditions and ad sources. All ads, regardless of source or labeling, were rated as effective as the 95% CIs of all measures did not overlap with the midpoint (4; neutral).

**Table 2.** Perceived Message Effectiveness Measures for AI-Generated Ads and Existing Ads by Official Health Agencies Across Labeling Conditions

Variable	Perceived message effectiveness score, mean (SD)^a^			
	No label	AI label	Health authority label	Combined label
AI-generated ads				
Effects perceptions measures^b^				
Vaping perception	2.59 (1.66)	2.63 (1.57)	2.47 (1.59)	2.75 (1.71)
Behavioral intent	2.65 (1.61)	2.73 (1.57)	2.62 (1.59)	2.69 (1.55)
Ad perceptions measures^c^				
Attention	4.64 (1.78)	4.62 (1.65)	4.79 (4.67)	4.75 (1.75)
Information	4.90 (1.69)	4.72 (1.59)	4.89 (1.63)	4.84 (1.70)
Convincingness	4.91 (1.73)	4.77 (1.63)	4.91 (1.67)	4.86 (1.73)
Existing ads				
Effects perceptions measures^b^				
Vaping perception	2.68 (1.69)	2.73 (1.56)	2.53 (1.59)	2.84 (1.75)
Behavioral intent	2.74 (1.62)	2.83 (1.57)	2.69 (1.60)	2.79 (1.58)
Ad perceptions measures^c^				
Attention	4.50 (1.80)	4.47 (1.71)	4.63 (1.73)	4.62 (1.79)
Information	4.77 (1.76)	4.63 (1.67)	4.77 (1.71)	4.72 (1.76)
Convincingness	4.73 (1.77)	4.60 (1.70)	4.73 (1.74)	4.66 (1.80)

Abbreviation: AI, artificial intelligence.

^a^ All measures used 7-point scales.

^b^ For effects perceptions, lower scores indicate better message effectiveness. Vaping perception: “This ad makes me think vaping is a very bad/good idea.” Behavioral intent: “This ad strongly discouraged/encouraged me from vaping.”

^c^ For ad perceptions, higher scores indicate better message effectiveness. Attention: “This ad grabbed my attention.” Information: “This ad provided useful information.” Convincingness: “This ad was convincing.”

#### Source Effects

Table 3 shows the results from the linear mixed model analysis. Overall, AI-generated ads were noninferior across all 5 measures compared with existing ads. Indeed, AI-generated ads had significantly better rating for 4 of the 5 measures, with small effect sizes ranging from 0.09 to 0.18 points on the 7-point scale.

**Table 3.** Results From the Linear Mixed Models Examining the Effects of Source Labeling, Ad Source, and Message Theme on Perceived Message Effectiveness for Full Sample (N = 614)^a^

Variable	b (95% CI)				
	Effects perceptions^b^		Ad perceptions^c^		
	Vaping perception	Behavioral intent	Attention	Information	Convincingness
Labeling					
Health authority label	0 [Reference]	0 [Reference]	0 [Reference]	0 [Reference]	0 [Reference]
AI label	0.18 (−0.11 to 0.47)	0.13 (−0.14 to 0.4)	−0.15 (−0.42 to 0.11)	−0.16 (−0.42 to 0.1)	−0.14 (−0.4 to 0.13)
Combined label	0.30 (0.01 to 0.59)^d^	0.09 (−0.18 to 0.36)	−0.01 (−0.28 to 0.25)	−0.05 (−0.3 to 0.21)	−0.06 (−0.32 to 0.21)
No label	0.13 (−0.16 to 0.43)	0.04 (−0.24 to 0.32)	−0.14 (−0.41 to 0.13)	−0.01 (−0.27 to 0.25)	−0.01 (−0.28 to 0.26)
Source					
AI generated	0 [Reference]	0 [Reference]	0 [Reference]	0 [Reference]	0 [Reference]
Existing ads	0.09 (0.01 to 0.16)^d^	0.09 (0.01 to 0.17)^d^	−0.15 (−0.26 to −0.03)^d^	−0.12 (−0.26 to 0.02)	−0.18 (−0.3 to −0.07)^d^
Theme					
Addiction	0 [Reference]	0 [Reference]	0 [Reference]	0 [Reference]	0 [Reference]
Financial impact	0.19 (0.07 to 0.31)^d^	0.16 (0.04 to 0.29)^d^	−0.26 (−0.44 to −0.08)^d^	−0.12 (−0.34 to 0.1)	−0.19 (−0.37 to −0.02)^d^
Health consequences	−0.11 (−0.23 to 0.01)	−0.11 (−0.23 to 0.01)	0.09 (−0.09 to 0.27)	0.17 (−0.05 to 0.39)	0.11 (−0.07 to 0.29)
Industry manipulation	0.21 (0.09 to 0.32)^d^	0.18 (0.06 to 0.3)^d^	−0.17 (−0.35 to 0.01)	−0.21 (−0.43 to 0.01)	−0.25 (−0.43 to −0.08)^d^
Social norm	0.32 (0.20 to 0.44)^d^	0.34 (0.22 to 0.46)^d^	−0.41 (−0.59 to −0.23)^d^	−0.36 (−0.58 to −0.14)^d^	−0.45 (−0.63 to −0.28)^d^
Intercept	2.34 (2.11 to 2.56)	2.49 (2.28 to 2.71)	4.92 (4.69 to 5.16)	4.99 (4.74 to 5.24)	5.07 (4.83 to 5.30)
Likelihood ratio test against model without conditions, χ^2^	4.19	0.1	2.09	1.97	1.33
* P* value	.24	.81	.55	.58	.72

Abbreviations: AI, artificial intelligence; b, mean difference.

^a^ All measures used 7-point scales.

^b^ For effects perceptions, lower scores indicate better ad effectiveness. Vaping perception: “This ad makes me think vaping is a very bad/good idea.” Behavioral intent: “This ad strongly discouraged/encouraged me from vaping.”

^c^ For ad perceptions, higher scores indicate better ad effectiveness. Attention: “This ad grabbed my attention.” Information: “This ad provided useful information.” Convincingness: “This ad was convincing.”

^d^ *P* < .05.

For effects perceptions (where lower scores indicate better effectiveness), existing ads had higher scores than AI-generated ones (mean difference [b] = 0.09 [95% CI, 0.01 to 0.16] for vaping perception and b = 0.09 [95% CI, 0.01 to 0.17] for behavioral intent), indicating worse effectiveness. For ad perceptions (where higher scores indicate better effectiveness), existing ads received significantly lower ratings for attention (b = −0.15 [95% CI, −0.26 to −0.03]) and convincingness (b = −0.18 [95% CI, −0.30 to −0.07]), indicating worse effectiveness (all *P* for noninferiority tests <.001). The effect on informativeness showed a similar pattern but did not reach statistical significance (b = −0.12 [95% CI, −0.26 to 0.02]).

#### Labeling Effects

Likelihood ratio tests comparing models with and without labeling conditions revealed no significant association of source labeling with any of the 5 PME measures (χ^2^ values ranging from 0.10 to 4.19; all *P* > .20). Results from the sensitivity analyses, which was limited to only the 462 participants (75%) who passed the manipulation check, produced similar results (eTable in Supplement 1).

## Discussion

This randomized clinical trial demonstrated that AI-generated ads were not only noninferior to existing ads from official health agencies but also performed slightly better on 4 of the 5 PME measures. We also found that source labeling had no significant association with any PME among young people. These findings suggest that AI-generated ads, developed through codesign with young people, can match or exceed the effectiveness of traditional campaign ads while maintaining credibility regardless of source attribution. These results have important implications for how health agencies might leverage AI technology while maintaining message credibility.

The effectiveness of the AI-generated ads can be attributed to 2 key factors: (1) the sophisticated capabilities of current GenAI systems that enable rapid generation and iteration of diverse content at scale, and (2) the systematic codesign process with young people. GenAI’s ability to simultaneously produce thousands of variations in text and image components allows for extensive experimentation with different themes, styles, and cultural adaptations. Through iterative feedback from focus groups and semistructured interviews, young people guided the refinement of our prompting strategies and design choices, which allowed for quick modifications and testing of multiple versions until the ads resonated with our target audience. This rapid iteration and scaling capability, combined with target audience input, represents a major advancement over traditional content development approaches.

The absence of an AI bias effect in our study differs from previous research that has generally found negative perceptions toward AI-generated content when the source is disclosed. These divergent findings can potentially be explained by differences in sampling and timing. Our study specifically focused on young people, whereas previous studies examined broader age ranges.^5^ Young people are often early adopters of new technologies and can develop proficiency in their use much faster than older generations. Since ChatGPT’s launch in November 2022, there have been numerous reports indicating that young people readily adopt GenAI technology.^28^ This greater familiarity and comfort with GenAI technology among young people may explain why our participants did not exhibit the negative bias toward AI-labeled ads.

The finding that AI-generated ads were perceived as equally effective as those from health agencies suggests that GenAI may be a double-edged sword. GenAI cannot only be used to expedite the development of health campaign messages, it can also rapidly generate widespread and persuasive health disinformation. For example, Menz et al^29^ showed that in just 65 minutes, a publicly available large language model could generate 102 distinct and persuasive blog articles containing disinformation about vaccines and vaping. These articles included fabricated patient and clinician testimonials and were strategically tailored for diverse demographic groups, such as young adults, parents, older persons, pregnant people, and those with chronic health conditions. The ease and speed with which convincing health disinformation can be mass-produced highlights the urgent need for increased public awareness of AI-generated content and robust regulatory frameworks that ensure transparency in the use of GenAI technologies.

### Limitations

This study is not without limitations. First, potential selection bias exists in our comparison materials. Although our AI-generated ads underwent iterative refinement through youth feedback, existing ads from official health agencies were selected using predetermined criteria, with unknown youth involvement in their development process. Although our selection criteria helped mitigate potential bias, the superior effectiveness of AI-generated ads might partly reflect our selection of comparison materials rather than solely representing the advantages of the AI-codesign approach. Second, despite PME being a validated and well-established predictor of behavior change,^25^ we cannot yet confirm whether these ads will actually discourage vaping behavior. Third, our nonprobability sample might not be representative of the population. Future research is needed to test generalizability of our findings.

## Conclusions

In this randomized clinical trial of vaping awareness social media ads, we demonstrated the potential of combining GenAI with youth codesign to develop effective vaping awareness ads. The findings revealed that AI-generated ads achieved PME scores that were noninferior to, and in most cases exceeded, those for existing ads from official health agencies. Labeling the ads as AI-generated or created by an official health agency had no significant association with PME among young people. Together, these findings suggested that GenAI combined with target population codesign approaches could enable health agencies to respond more quickly to emerging health challenges.

## Notes

Supplement 1. **eTable.** Results from the linear mixed models examining the effects of source labelling, ad source, and message theme on perceived message effectiveness (PME) among participants who passed the manipulation check (N = 462)

Supplement 2.
Data Sharing Statement
