# Supplementary material for: Generative Artificial Intelligence With Youth Codesign to Create Vaping Awareness Advertisements
Source: JAMA Netw Open. 2025 Jul 31;8(7):e2514040. doi: 10.1001/jamanetworkopen.2025.14040 (PMC12314720; doi:10.1001/jamanetworkopen.2025.14040)
Supplement: Supplement 1. — eTable. Results from the linear mixed models examining the effects of source labelling, ad source, and message theme on perceived message effectiveness (PME) among participants who passed the manipulation check (N = 462) [file jamanetwopen-e2514040-s001.pdf]

Supplemental Online Content

Leung J, Sun T, Stjepanović D, et al. Generative artificial intelligence with youth codesign to create effective vaping awareness advertisements. *JAMA Netw Open*. 2025;8(7):e2514040. doi:10.1001/jamanetworkopen.2025.14040

eTable. Results from the linear mixed models examining the effects of source labelling, ad source, and message theme on perceived message effectiveness (PME) among participants who passed the manipulation check (N = 462)

This supplemental material has been provided by the authors to give readers additional information about their work.

© 2025 Leung J et al. *JAMA Network Open*.

eTable. Results from the linear mixed models examining the effects of source labelling, ad source, and message theme on perceived message effectiveness (PME) among participants who passed the manipulation check (*N* = 462).

	**Effects perceptions‡**				**Ad perceptions§**					
	**1) Vaping perception**		**2) Behavioural intent**		**3) Attention**		**4) Information**		**5) Convincingness**	
	**b**	**95% CI**	**b**	**95% CI**	**b**	**95% CI**	**b**	**95% CI**	**b**	**95% CI**
**Labelling**										
**(Ref: Health Authority Label)**										
AI label	0.20	(-0.09, 0.49)	0.08	(-0.2, 0.36)	-0.14	(-0.42, 0.13)	-0.14	(-0.41, 0.13)	-0.12	(-0.41, 0.16)
Combined label	0.39*	(0.08, 0.69)	0.11	(-0.19, 0.4)	-0.02	(-0.31, 0.27)	-0.04	(-0.33, 0.24)	-0.07	(-0.37, 0.22)
No label	0.10	(-0.22, 0.42)	-0.02	(-0.33, 0.28)	-0.10	(-0.4, 0.21)	0.00	(-0.3, 0.29)	-0.04	(-0.35, 0.27)
**Source (Ref: AI generated)**										
Existing ads	0.09*	(0.01, 0.18)	0.11*	(0.02, 0.19)	-0.18*	(-0.31, -0.05)	-0.12	(-0.29, 0.04)	-0.21*	(-0.35, -0.08)
**Theme (Ref: Addiction)**										
Financial impact	0.22*	(0.09, 0.36)	0.19*	(0.05, 0.32)	-0.29*	(-0.5, -0.08)	-0.11	(-0.37, 0.16)	-0.24*	(-0.45, -0.03)
Health consequences	-0.11	(-0.24, 0.03)	-0.10	(-0.24, 0.03)	0.08	(-0.13, 0.29)	0.20	(-0.06, 0.46)	0.12	(-0.09, 0.33)
Industry manipulation	0.23*	(0.10, 0.37)	0.22*	(0.08, 0.35)	-0.21	(-0.42, 0)	-0.24	(-0.51, 0.02)	-0.32	(-0.53, -0.11)
Social norm	0.37*	(0.24, 0.50)	0.40*	(0.27, 0.54)	-0.48*	(-0.68, -0.27)	-0.40*	(-0.67, -0.14)	-0.55*	(-0.76, -0.34)
**Intercept**	2.18	(1.96, 2.42)	2.39	(2.16, 2.61)	4.98	(4.72, 5.23)	4.97	(4.69, 5.25)	5.12	(4.86, 5.38)
**Likelihood ratio test against model without conditions**	**χ^2**	**p-value**	**χ^2**	**p-value**	**χ^2**	**p-value**	**χ^2**	**p-value**	**χ^2**	**p-value**
	6.65	0.08	0.99	0.80	1.26	0.74	1.32	0.72	0.78	0.85

Note: All measures used 7-point scales: ‡Effects Perceptions: Lower scores indicate better ad effectiveness. (1) Vaping Perception: "This ad makes me think vaping is a very bad/good idea". (2) Behavioural Intent: "This ad strongly discouraged/encouraged me from vaping". §Ad Perceptions (3-5): Higher scores indicate better ad effectiveness. (3) Attention: "This ad grabbed my attention", (4) Information: "This ad provided useful information", (5) Convincingness: "This ad was convincing". *$p < 0.05$

© 2025 Leung J et al. *JAMA Network Open*.
