# Supplementary material for: Generative Artificial Intelligence With Youth Codesign to Create Vaping Awareness Advertisements
Source: JAMA Netw Open. 2025 Jul 31;8(7):e2514040. doi: 10.1001/jamanetworkopen.2025.14040 (PMC12314720; doi:10.1001/jamanetworkopen.2025.14040)
Supplement: Supplement 2. — Data Sharing Statement [file jamanetwopen-e2514040-s002.pdf]

Data Sharing Statement

Leung. Generative Artificial Intelligence With Youth Codesign to Create Vaping Awareness Advertisements. *JAMA Netw Open*. Published July 29, 2025.
doi:10.1001/jamanetworkopen.2025.14040

Data
Data available: Yes
Data types: Data dictionary
How to access data: https://github.com/gckc123/AIvaping
When available: With publication

Supporting Documents
Document types: None

Additional Information
Who can access the data: Publicly available
Types of analyses: N/A
Mechanisms of data availability: N/A
